# Effect of changes in the arm physical parameters on the minimum torque-change trajectories of human reaching movements

**DOI:** 10.1007/s11571-026-10428-0

**Published:** 2026-03-04

**Authors:** Kotaro Muramatsu, Takahiro Kagawa, Naomichi Ogihara

**Affiliations:** 1 Laboratory of Human Evolutionary Biomechanics, Department of Biological Sciences, Graduate School of Science, The University of Tokyo, 7-3-1 Hongo, Bunkyo-ku, 113-0033 Tokyo, Japan; 2 Department of Mechanical Engineering, Aichi Institute of Technology, 1247 Yachigusa, Yakusa-cho, Toyota, 470-0392 Aichi, Japan

**Keywords:** Minimum torque-change model, Reaching movement, Dynamic optimization, Arm model, Hand trajectory

## Abstract

The minimum torque-change model is a computational model describing the trajectory formation of the point-to-point reaching movement in humans. This model roughly predicts a straight hand trajectory with a bell-shaped velocity profile, as observed in human reaching movements. However, the minimum torque-change criterion is a dynamic quantity, and the calculated trajectories could be, at least to some extent, affected by changes in the arm’s physical parameters such as mass, moment of inertia, and viscosity of each link. This study systematically investigates how changes in the arm’s physical parameters affect the optimal arm trajectories calculated based on the minimum torque-change criterion. The calculated optimal trajectories were largely curved, particularly when the physical parameters of the forearm were doubled or halved from the original physical parameters. Furthermore, when the original parameters were modified to be biomechanically more appropriate, the trajectories were also largely curved, unlike those in actual human reaching movements. The results suggest that the hand trajectory in human reaching movements may be determined by a dynamic optimization criterion that is less sensitive to variations in the biomechanical properties.

**Supplementary Information:**

The online version contains supplementary material available at 10.1007/s11571-026-10428-0.

## Introduction

Voluntary movements, such as point-to-point reaching movements in humans, are realized by the coordinated activation of muscles spanning the joints in the musculoskeletal system. Kinematically, infinitely possible trajectory patterns can be followed to move the hand from a particular starting point to an end point. However, human reaching movements exhibit invariant features; for instance, the neurotypical human hand trajectories are often roughly straight (i.e., exhibiting little curvature), and the velocity profiles are bell-shaped (Morasso 1981). Therefore, researchers have hypothesized that the trajectories of human reaching movements are computed in the central nervous system (CNS) ahead of the actual motion generation by minimizing a certain objective criterion, such as hand position jerk (time derivative of the hand acceleration) (Flash and Hogan 1985), joint angle jerk (Rosenbaum et al. 1995), joint torque rate of change (Uno et al. 1989), muscle force rate of change (Dornay et al. 1996), motor command rate of change (Kawato 1992), end point variance (Harris and Wolpert 1998), and energetic costs (Nishii and Taniai 2009; Taniai and Nishii 2015). Subsequently, reaching movements are generated using a feedforward control based on optimally planned trajectories. Recently, optimal feedback control theory has been regarded as a more biologically feasible computational framework for generating human reaching movement (Todorov and Jordan 2002; Shadmehr and Krakauer 2008; Qian et al. 2013). Optimal feedback control explains movement as choosing a feedback control policy that minimizes an expected cost in the presence of motor and sensory noise; the observed trajectories then emerge from the interaction between this policy and the task and body dynamics. Importantly, regardless of whether a model is formulated as feedforward trajectory planning or as feedback policy optimization, it should ultimately be evaluated by the movement patterns it predicts. Because these predictions depend critically on the optimization criterion (i.e., the cost function), it is informative to examine how different criteria shape the resulting trajectories. In the present study, we do not analyze optimal feedback control; instead, we focus on trajectory-level predictions obtained from feedforward objective criteria.

The minimum hand jerk model (Flash and Hogan 1985) is a kinematic criterion in extrinsic coordinates based on hand position that predicts strictly straight movement trajectories for point-to-point reaching. The minimum angle jerk model (Rosenbaum et al. 1995) differs in utilizing intrinsic angle coordinates, while it shares the planning criterion in a kinematic space. Because such kinematics-based criteria do not explicitly incorporate arm dynamics, their trajectory predictions are expected to be relatively insensitive to changes in limb mechanics or external loading. In multi-joint reaching, however, trajectories can be influenced by arm dynamics (e.g., interaction torques), motivating criteria formulated in a dynamics-based space. A classic example is the minimum torque-change model (Uno et al. 1989), which favors smooth joint-torque profiles. Consequently, generating trajectories from this class of models requires specifying physical parameters of the arm, and the predicted hand trajectories can depend on those parameters.

Flash (1990) and Nakano et al. (1999) previously noted that the minimum torque-change trajectory was sensitive to the changes in the physical parameters of the arm model. However, these studies did not demonstrate how large the minimum torque-change trajectories could be deviated from the measured hand trajectories by the alteration of the arm’s physical parameters (masses and moments of inertia of the upper arm and forearm segments and viscous property of the shoulder and elbow joints) in a systematic manner. Therefore, the fact that the minimum torque-change trajectory was quite sensitive to the changes in the physical parameters of the arm model has been commonly overlooked, and the model has been generally regarded as being capable of reproducing the characteristic hand trajectories observed in human reaching movements without any undue influence of the selection of the arm’s physical parameters. This lack of clarity may also limit the applicability of the minimum torque-change model to more complex tasks, such as whole-body reaching (Kudo et al. 2016), underscoring the importance of systematically examining the parameter sensitivity. Following the minimum torque-change model, several extensions have been proposed, including the minimum muscle-tension change model (Dornay et al. 1996) and the minimum motor command change model (Kawato 1992). There also exists a proposed model, the minimum commanded torque-change model (Nakano et al. 1999; Wada et al. 2001), the essence of which lies in incorporating compensatory components into the commanded torques to attenuate torque attenuation due to muscle viscosity. Overall, while these refined models can help predict trajectories more closely to experiments and capture physiological plausibility, they may still share the unresolved issue of sensitivity to the arm’s physical parameters, as noted above, particularly regarding its dynamic and mechanical properties.

The present study aims to explore, as a problem-posing approach, how changes in the arm’s physical parameters affect trajectories derived from such intrinsic-dynamic criteria, rather than to compare their ability to reproduce experimental movements. To this end, we adopt the minimum torque-change model not to adjudicate its overall validity, but to use it as a minimal dynamics-dependent testbed in which the influence of biomechanical parameters on trajectory-level predictions can be isolated and quantified; it is also the most fundamental member of this class, still influential in contemporary motor control studies, and relatively inexpensive to compute. Specifically, we systematically investigate how alterations in the arm’s physical parameters, such as masses and moments of inertia of the segments and the viscous properties of the joints, affect the hand trajectories calculated based on this model. Although the original work by Uno et al. (1989) showed that the minimum torque-change model can generate roughly straight hand trajectories, its parameter settings were reported to be inappropriate (Nakano et al. 1999), suggesting that this property may be specific to certain parameter configurations. To examine whether this trajectory-generating property remains robust, we tested the hypothesis that changes in the arm’s physical parameters have only a minor effect on the predicted hand trajectories, regardless of their type and position, as holding an object with a hand or wearing an elbow supporter appears to have virtually no effect on the generated hand trajectories. Characterizing parameter sensitivity is important for prediction and adaptation whenever limb parameters must be assumed or estimated, including contemporary extensions such as the minimum commanded torque-change model and feedback-based frameworks that rely on internal models of arm dynamics.

## Methods

### Two-link arm model

**Fig. 1:**
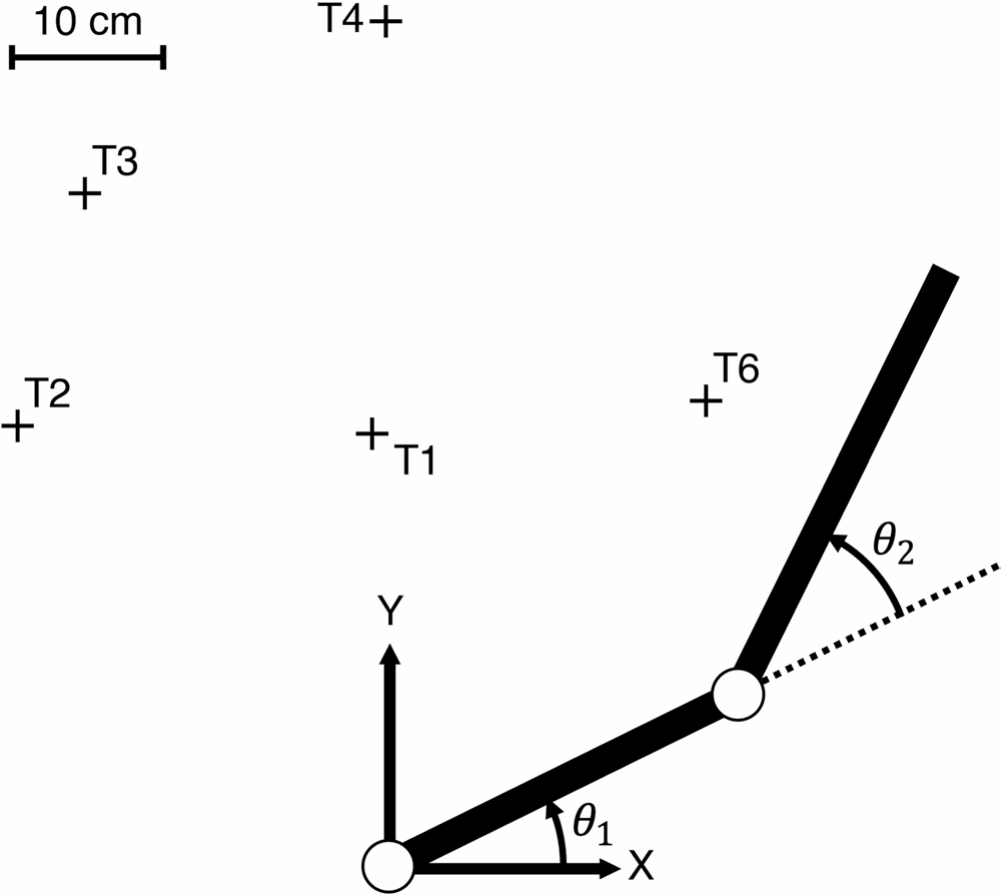
A two-link human arm model in a horizontal plane comprising an upper arm and forearm. The origin of the horizontal workspace corresponds to the shoulder joint, and the five target positions are set according to Uno et al. (1989). The shoulder and elbow joint angles are represented as ${\theta}_{1}$ and ${\theta}_{2}$, respectively

Trajectory generation was based on a two-link model of the upper arm and forearm in a horizontal plane shown in Fig. 1. We set the origin of the horizontal workspace to the shoulder joint, and the target positions were set according to the original paper (Uno et al. 1989). The equations of motion of the two-link arm model can be derived as follows:

1 $$\begin{array}{c}\dot{\boldsymbol{q}}=f\left(\boldsymbol{q},\boldsymbol{\tau}\right)=\left[\begin{array}{c}\dot{\boldsymbol{\theta}}\\{M\left(\boldsymbol{\theta}\right)}^{-1}\left(\boldsymbol{\tau}-\boldsymbol{B}\left(\boldsymbol{\theta},\dot{\boldsymbol{\theta}}\right)\right)\end{array}\right],\end{array}$$

with

$$M\left(\boldsymbol{\theta}\right)=\left[\begin{array}{cc}{I}_{1}+{I}_{2}+{m}_{2}{{L}_{1}}^{2}+2{m}_{2}{L}_{1}{r}_{2}\mathrm{c}\mathrm{o}\mathrm{s}{\theta}_{2}&{I}_{2}+{m}_{2}{L}_{1}{r}_{2}\mathrm{c}\mathrm{o}\mathrm{s}{\theta}_{2}\\{I}_{2}+{m}_{2}{L}_{1}{r}_{2}\mathrm{c}\mathrm{o}\mathrm{s}{\theta}_{2}&{I}_{2}\end{array}\right],$$

$$\boldsymbol{B}\left(\boldsymbol{\theta},\dot{\boldsymbol{\theta}}\right)=\left[\begin{array}{c}-{m}_{2}{L}_{1}{r}_{2}\left(2{\dot{\theta}}_{1}+{\dot{\theta}}_{2}\right){\dot{\theta}}_{2}\mathrm{s}\mathrm{i}\mathrm{n}{\theta}_{2}\\{m}_{2}{L}_{1}{r}_{2}{{\dot{\theta}}_{1}}^{2}\mathrm{s}\mathrm{i}\mathrm{n}{\theta}_{2}\end{array}\right]+\left[\begin{array}{c}{b}_{1}{\dot{\theta}}_{1}\\{b}_{2}{\dot{\theta}}_{2}\end{array}\right],$$

where $$\boldsymbol{\theta}={\left[\begin{array}{cc}{\theta}_{1}&{\theta}_{2}\end{array}\right]}^{\boldsymbol{\top}}$$ is the vector containing the shoulder and elbow joint angles, $\dot{\boldsymbol{\theta}}$ is the time derivative of $\boldsymbol{\theta}$, $$\boldsymbol{q}={\left[\begin{array}{cc}{\boldsymbol{\theta}}^{\boldsymbol{\top}}&{\dot{\boldsymbol{\theta}}}^{\boldsymbol{\top}}\end{array}\right]}^{\boldsymbol{\top}}$$ is a combined state vector, and $$\boldsymbol{\tau}={\left[\begin{array}{cc}{\tau}_{1}&{\tau}_{2}\end{array}\right]}^{\boldsymbol{\top}}$$ is a vector of the shoulder and elbow joint torques. Moreover, ${L}_{i}$, ${r}_{i}$, ${m}_{i}$, ${I}_{i}$, and $\:{b}_{i}$ are the length, position of the center-of-mass from the proximal joint, mass, moment of inertia around the proximal joint, and viscous (or damping) coefficient, respectively, of the $i$-th segment or joint ($i=1,2$ correspond to the upper arm and forearm, or the shoulder and elbow joints, respectively).= Based on the parallel axis theorem, ${I}_{i}={I}_{Gi}+{m}_{i}{{r}_{i}}^{2}$, where ${I}_{Gi}$ is the moment of inertia around the center-of-mass of the $i$-th segment. The values of the original physical parameters adopted by Uno et al. (1989) are shown in the upper row of Table 1.                

**Table 1:** Comparison between the original physical parameter values of Uno et al. (1989) and the new, biomechanically relevant ones introduced in this study, which were estimated based on Winter (2009)

	Length [m]		Center-of-mass [m]		Mass [kg]		Moment of inertia [kg m^2^]		Viscosity [*N* m s/rad]	
	${L}_{1}$		${r}_{1}$	${r}_{2}$	${m}_{1}$	${m}_{2}$	${I}_{1}$(${I}_{G1}$)	${I}_{2}$(${I}_{G2}$)	${b}_{1}$	${b}_{2}$
Original parameters by Uno et al. (1989)	0.25	0.35	0.11	0.15	0.9	1.1	0.065 (0.054)	0.100 (0.075)	0.08	0.08
Biomechanically relevant parameters by Winter (2009)	0.25	0.35	0.11	0.15	1.82	1.43	0.0334 (0.0118)	0.0487 (0.0160)	0.0, 0.2, 0.4, 0.8	equal to ${b}_{1}$

### Calculation of minimum torque-change trajectories

The minimum torque-change trajectory between a given start and end point can be calculated by solving the following minimization problem of the objective function $J$ (an integral of squared torque changes during movement time $T$) under the constraints of the initial condition, terminal condition, and equation of motion (Eq. 1) as:

2 $${\mathrm{minimize~~~}}J = \mathop \smallint \limits_{0}^{T} \frac{1}{2}\left\| {\user2{\dot{\tau }}} \right\|^{2} dt,$$

3 $$\begin{array}{c}\text{subject to}\:\left\{\begin{array}{c}\boldsymbol{q}\left(0\right)={\boldsymbol{q}}_{0},\boldsymbol{\tau}\left(0\right)={\boldsymbol{\tau}}_{0}\\\boldsymbol{q}\left(T\right)={\boldsymbol{q}}_{T},\boldsymbol{\tau}\left(T\right)={\boldsymbol{\tau}}_{T}\\\dot{\boldsymbol{q}}=\boldsymbol{f}\left(\boldsymbol{q},\boldsymbol{\tau}\right)\end{array}\right.,\end{array}$$

where ${\boldsymbol{q}}_{0}$ and ${\boldsymbol{q}}_{T}$ are the state vectors of the joint angles and angular velocities at the hand start and end points represented in the joint coordinate system, respectively (the time is zero and $T$, respectively), ${\boldsymbol{\tau}}_{0}$ and ${\boldsymbol{\tau}}_{T}$ are the joint torque vectors when the hand is at the start and end points (${\boldsymbol{\tau}}_{0}={\boldsymbol{\tau}}_{T}=0$), respectively. By solving this problem using a variational method and a Newton-like method, following the calculation described by Uno et al. (1989), the minimum torque-change trajectories can be computed in the state space. We refer to this procedure as the “method I.”    

In regions with sufficiently high viscosity, such as ${b}_{i}=0.4$ and $0.8$ N m s/rad, the optimization method I described above becomes difficult to converge, even after prolonged computation. To address this issue, we used two alternative annealing methods II and III. In these approaches, we introduced a new evaluation function ${\bar{J}}$ consisting of the objective function (Eq. 2) scaled by a factor , combined with a penalty function $\Phi$:

4 $$\bar{J} = \lambda J + \Phi.$$

In both annealing methods, $\lambda$ was gradually decreased from a sufficiently large value, and at each step, the function (Eq. 4) was minimized via gradient descent. The essential difference between the two annealing methods is the way to obtain the gradient of ${\bar{J}}$. The first annealing (method II) numerically differentiates ${\bar{J}}$ with respect to the torque profiles $\boldsymbol{\tau}$ (as adopted in Nakano et al. 1999) by applying small perturbation $\varepsilon$. The gradient of ${\bar{J}}$ for the torque of joint $k$ (elbow or shoulder) at time ${t}_{i}$ is given by

5 $$\frac{{\partial \bar{J}}}{{\partial \tau _{k} \left( {t_{i} } \right)}} = \frac{{\bar{J}\left( {\tau _{k} \left( {t_{i} } \right) + \varepsilon } \right) - \bar{J}\left( {\tau _{k} \left( {t_{i} } \right)} \right)}}{\varepsilon}.$$

Because $\boldsymbol{\tau}$ is to be optimized, the initial and terminal states at time ${t}_{i}=0$, $T$, are fixed according to Eq. 3, while the rest are updated by Eq. 5. The penalty function requires terminal constraints for $\boldsymbol{q}$ as follows:

6 $$\Phi = \frac{1}{2}\left\| {\user2{q}\left( T \right) - \user2{q}_{T} } \right\|^{2}.$$

In contrast, the second annealing (method III) derived the gradient of ${\bar{J}}$ with respect to the torque change profiles $\dot{\boldsymbol{\tau}}$ through a similar calculation procedure to Dornay et al. (1996). The gradient ${\bar{J}}$ for the torque change at time ${t}_{i}$ is calculated by recursive equations formulated by discretizing the dynamic equations (Eq. 1) using a Euler method. In addition to , the penalty function contains the terminal conditions for $\boldsymbol{\tau}$ because the optimization variables are $\dot{\boldsymbol{\tau}}$:

7 $$\Phi = \frac{1}{2}\left\| {\user2{q}\left( T \right) - \user2{q}_{T} } \right\|^{2} + \frac{1}{2}\left\| {\user2{\tau }\left( T \right) - \user2{\tau }_{T} } \right\|^{2} .$$

Both annealing methods achieved more stable convergence than the Newton-like method I. In case the results of the two annealing methods differed in trajectory shapes and values of the objective function (Eq. 2), we ultimately selected the trajectories with the smaller objective function values as the “minimum torque-change trajectories.”

Using all three methods described above, the five hand trajectory patterns shown in Fig. 1 were obtained, consistent with Uno et al. (1989). The movement duration $T$ was determined as 0.75 s for all trajectories, and the time step size ${\Delta}t$ was set to 0.001 s.        

### Changes in the physical parameters of the arm model

Our study addresses two related but distinct questions: (i) whether the “roughly straight” trajectories reported in Uno et al. (1989) are specific to their parameter set, and (ii) whether the key sensitivity findings persist under a biologically plausible baseline. Accordingly, we treat Uno’s parameter set as a historical reference baseline for question (i), not as a physiologically justified setting, and we use another parameter set derived from Winter (2009) as a biologically plausible baseline for question (ii).

To address question (i), we adopt a one-at-a-time design to quantify first-order effects and isolate how each parameter individually changes curvature while keeping all others fixed. Even though Uno et al. (1989) attempted to determine the original physical parameter values (shown in Table 1) based on measurement of human and monkey arms, some of these values have been reported as over- or underestimated (Nakano et al. 1999). To investigate how changes in the arm’s physical parameters affect the calculated trajectories, we (1) doubled and halved the masses of the segments (${m}_{1},{m}_{2}$), (2) doubled and halved the moments of inertia of the segments around the center-of-mass (${I}_{G1},{I}_{G2}$), and (3) quintupled and one-fifth viscous coefficients (${b}_{1},{b}_{2}$) in the original parameters and recalculated the hand trajectories. We used multiplicative perturbations (e.g., $\times2$ and $\times1/2$) so that increases and decreases are applied by the same factor around the baseline and can be compared directly (equivalently, symmetric changes on a log scale); this proportional scaling is also natural for body-size–related parameters and avoids nonsensical values that could arise under additive perturbations. The range of parameter variations considered here was chosen to be sufficiently large to include values that are potentially more biologically relevant than those originally used by Uno et al. (1989), and, for some parameters, to exceed the typical variation observed in the general population. Specifically, we chose the scaling factors so that the perturbed values, starting from Uno’s original parameters, would cover the range of Winter’s parameter set described below. These perturbations are intentionally large, especially compared with typical inter-individual variability reported for some parameters (e.g., the standard deviation of ${m}_{1}$ is 0.158 kg when body mass is assumed to be 65 kg; Zatsiorsky 2002).        

For question (ii), we further considered a biologically plausible baseline by replacing all the original physical parameter values with those estimated using anthropometric data based on cadavers (Winter 2009), in a similar approach to Tsuzuki and Ogihara (2018). The new physical parameter values are shown in the lower row of Table 1, which were calculated by assuming that the body mass of the model subject was 65 kg. The viscous coefficients (${b}_{1},{b}_{2}$) were changed from 0 to 0.2, 0.4, and 0.8 N m s/rad, to compensate for fundamental difficulties in the precise quantification of viscosity values. Note that we set ${b}_{1}$ and ${b}_{2}$ equal since the previous studies (Nakano et al. 1999; Tsuzuki and Ogihara 2018) have also specified both at comparable values.

### Evaluation of trajectory curvature

To evaluate each trajectory curvature, we introduce the whole deviation $W$, which represents the area between the reference straight line connecting the start and end points and the model-predicted trajectory. In Nakano et al. (1999), the whole deviation $W$ was defined as the signed area that can quantify the trajectory’s curving direction relative to the reference straight line, with positive or negative values of $W$ indicating a rightward or leftward curve, respectively. However, this approach cannot be applied to trajectories that curve in a sigmoidal manner. Therefore, we calculated the absolute value of the whole deviation. For details on the trajectory’s curvature and shape, we also refer to the visual trajectory display.    

## Results

### Effect of changes in the mass ${m}_{1},{m}_{2}$

**Fig. 2:**
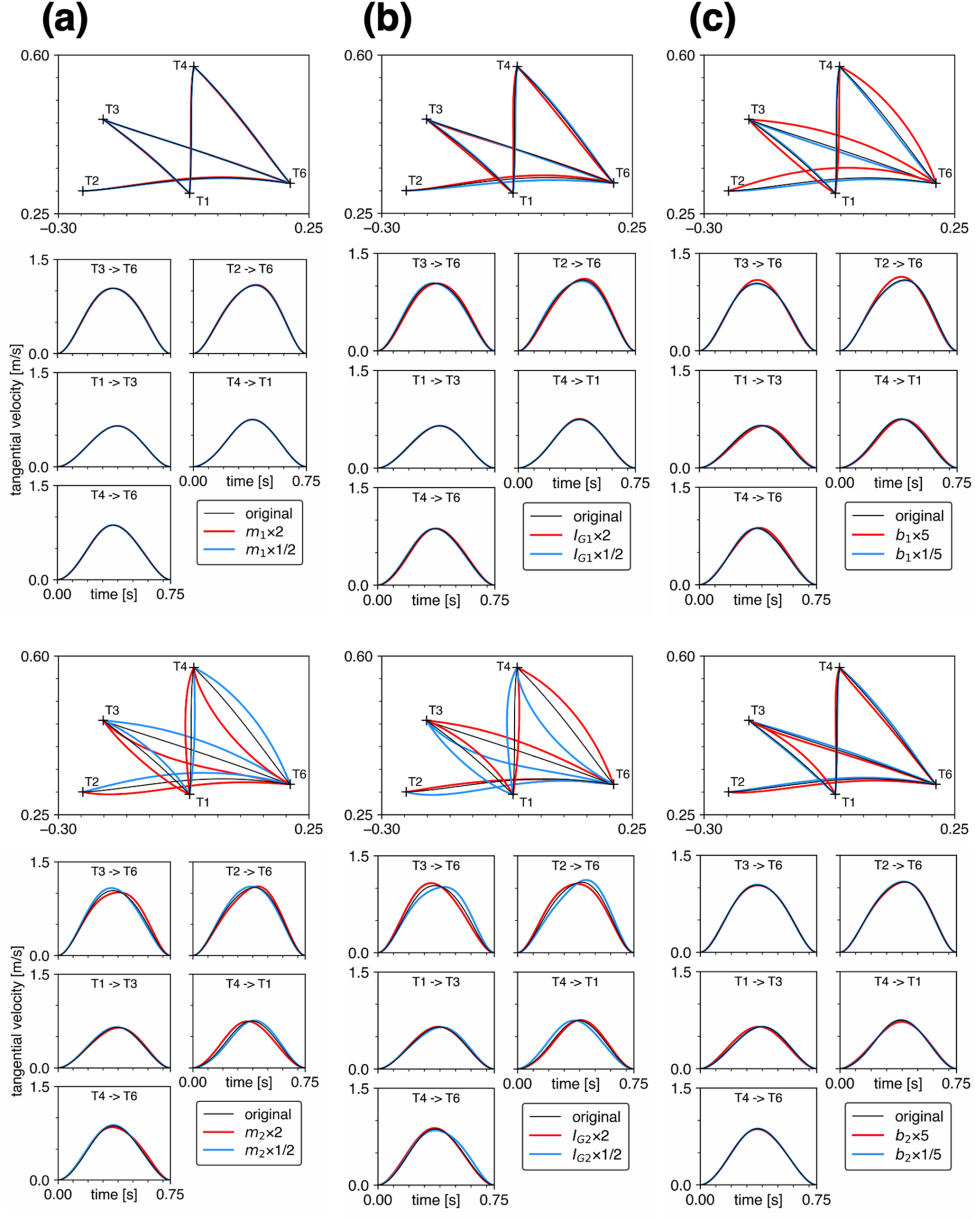
Comparisons of the minimum torque-change trajectories predicted by Uno’s original model (black) with those predicted when **a** the segment masses ${m}_{1}$ (top) and ${m}_{2}$ (bottom) were doubled (red) or the halved (cyan), **b** the segment moments of inertia ${I}_{G1}$ (top) and ${I}_{G2}$ (bottom) were doubled (red) or the halved (cyan), and **c** the viscous coefficients ${b}_{1}$ (top) and ${b}_{2}$ (bottom) were quintupled (red) or the one-fifth (cyan), one-at-a-time. Comparisons of the hand tangential velocities of the five trajectories are also included

**Fig. 3:**
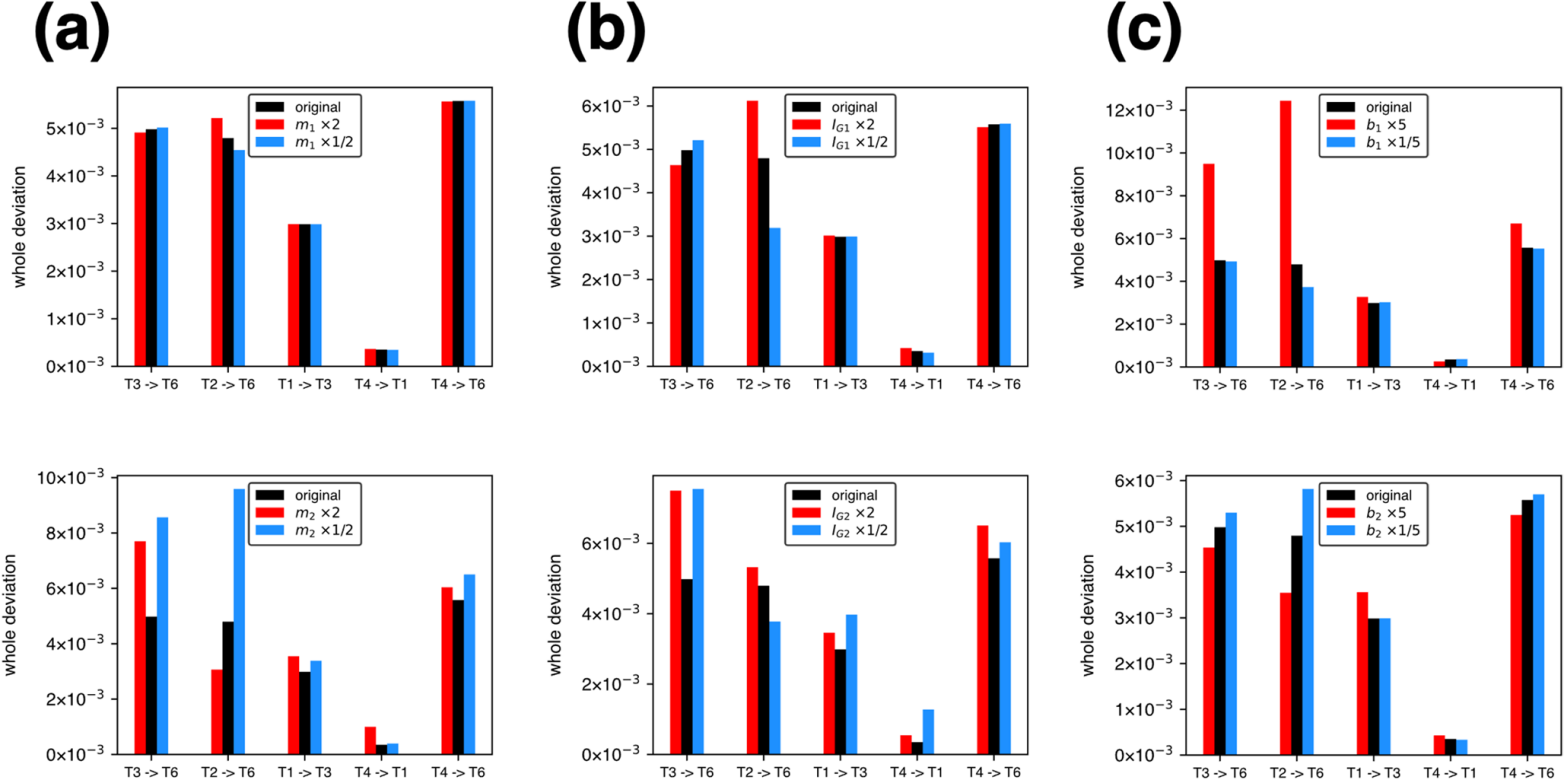
Comparison of the whole deviations of the original trajectories and all parameter-changed trajectories shown in Fig. 2; **a** the segment masses ${m}_{1}$ (top) and ${m}_{2}$ (bottom) were doubled (red) or the halved (cyan), **b** the segment moments of inertia ${I}_{G1}$ (top) and ${I}_{G2}$ (bottom) were doubled (red) or the halved (cyan), and **c** the viscous coefficients ${b}_{1}$ (top) and ${b}_{2}$ (bottom) were quintupled (red) or the one-fifth (cyan)

Figure 2a presents a comparison of the five minimum torque-change trajectories predicted based on Uno’s original parameters with those predicted when the segment mass was changed one-at-a-time. The trajectories predicted based on Uno’s original parameters were perfectly consistent with the trajectories presented in Fig. 3A-a of Uno et al. (1989), demonstrating that the minimum torque-change trajectories were successfully calculated. The whole deviation for each of the five trajectories under each parameter condition is shown in Fig. 3a. The changes in the mass of the upper arm (${m}_{1}$) had virtually no effect on the trajectories and velocity profiles (mean absolute change in whole deviation relative to baseline: 2.8% for doubling and 1.6% for halving across the five trajectories). However, the change in the mass of the forearm (${m}_{2}$) had a large effect on the trajectories and velocity profiles (corresponding values: 60.6% and 42.8%, respectively). The hand trajectories were more curved than the original roughly straight trajectories. Moreover, the trajectories tended to be concave or curved leftward when ${m}_{2}$ was doubled, and vice versa when ${m}_{2}$ was halved. The velocity profiles tended to be skewed, particularly when ${m}_{2}$ was doubled.      

### Effect of changes in the moment of inertia ${I}_{G1},{I}_{G2}$

Figure 2b compares the five minimum torque-change trajectories predicted based on Uno’s original parameters with those predicted when the segment moments of inertia were changed one-at-a-time. See also the whole deviations shown in Fig. 3b. The variation in the moment of inertia of the upper arm (${I}_{G1}$) had only a minor effect on the trajectories and velocity profiles (mean absolute change in whole deviation relative to baseline: 11.4% for doubling and 9.8% for halving across the five trajectories), whereas the change in the moment of inertia of the forearm (${I}_{G2}$) had a large effect on the trajectories and velocity profiles (corresponding values: 29.9% and 75.5%, respectively). The trajectories tended to be concave or curved leftward when ${I}_{G2}$ was halved, and vice versa when ${I}_{G2}$ was doubled. The velocity profiles tended to be skewed, particularly when ${I}_{G2}$ was halved. Therefore, the trend was opposite to that of ${m}_{2}$.    

### Effect of changes in the viscous coefficient ${b}_{1}, {b}_{2}$

Figure 2c presents a comparison of the five minimum torque-change trajectories predicted based on Uno’s original parameters with those predicted when the joint viscous coefficients were changed one-at-a-time. According to the whole deviations shown in Fig. 3c, the trajectories were largely affected when the shoulder viscous coefficient (${b}_{1}$) was quintupled (mean absolute change in whole deviation relative to baseline: 61.5% across the five trajectories). In contrast, the trajectories were not affected when the elbow viscous coefficient (${b}_{2}$) increased (corresponding values: 16.5%). The trajectories tended to be convex when ${b}_{1}$ was quintupled, except for the path from T1 to T3. Reducing the viscous coefficients to one-fifth of the initial value slightly affected the trajectories (corresponding values: 5.9% for reducing ${b}_{1}$ and 6.9% for ${b}_{2}$) and velocity profiles.        

To assess whether these main sensitivity patterns depend on the baseline parameter set, we additionally performed the same one-at-a-time perturbations around Winter’s parameter set (see Supplemental Materials). The key qualitative findings were reproduced, indicating that the pronounced sensitivity to specific parameters is not an artifact of Uno’s baseline.

### Biomechanically relevant physical parameters

**Fig. 4:**
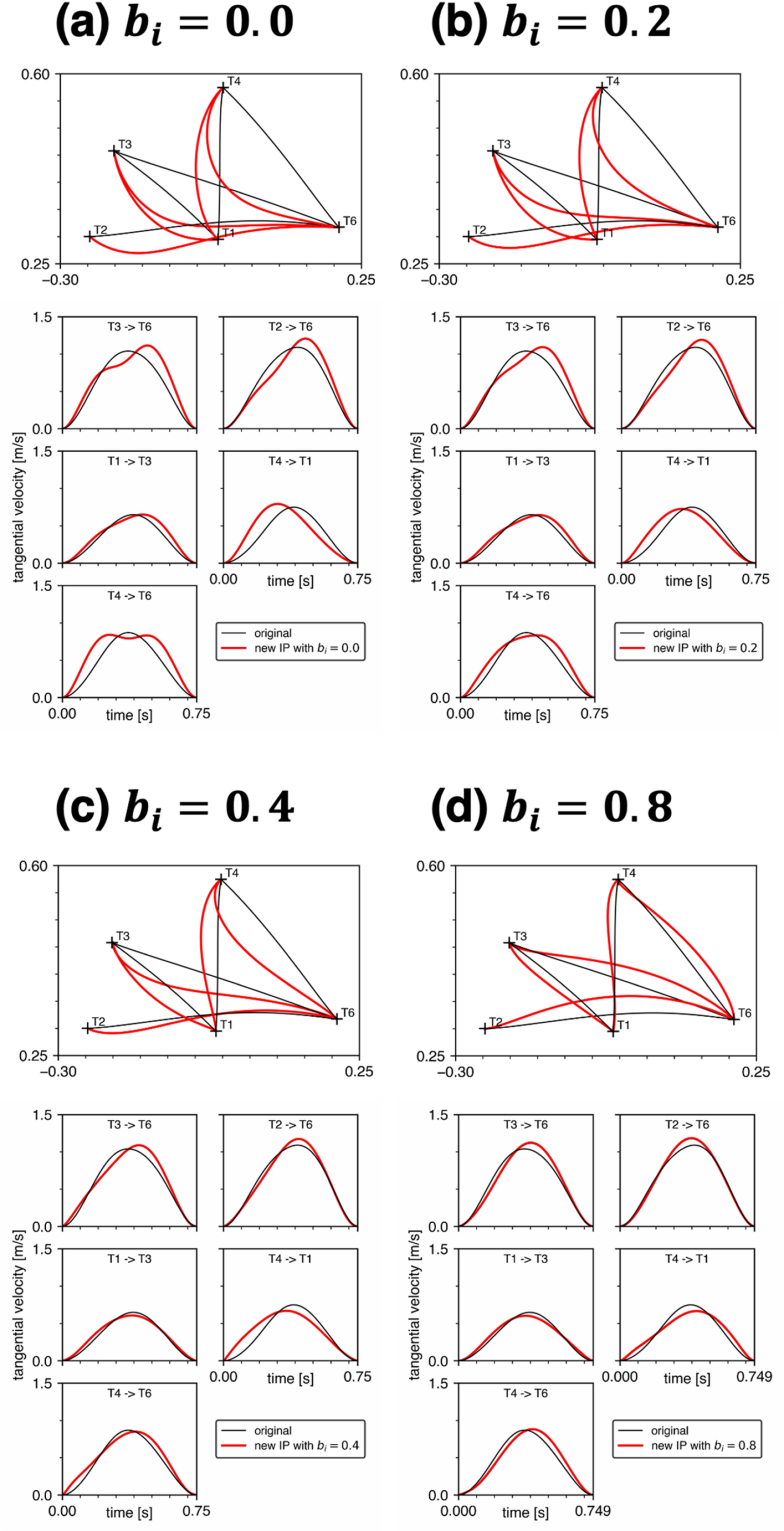
Comparisons of the minimum torque-change trajectories predicted by Uno’s original model (black) with those predicted when all inertial parameters (IP) were modified according to Winter’s biomechanically relevant physical parameters (red). The viscous coefficients ${b}_{1}$ and ${b}_{2}$ were equal and set as 0.0, 0.2, 0.4 or 0.8. Comparisons of the hand tangential velocities of the five trajectories are also included

**Fig. 5:**
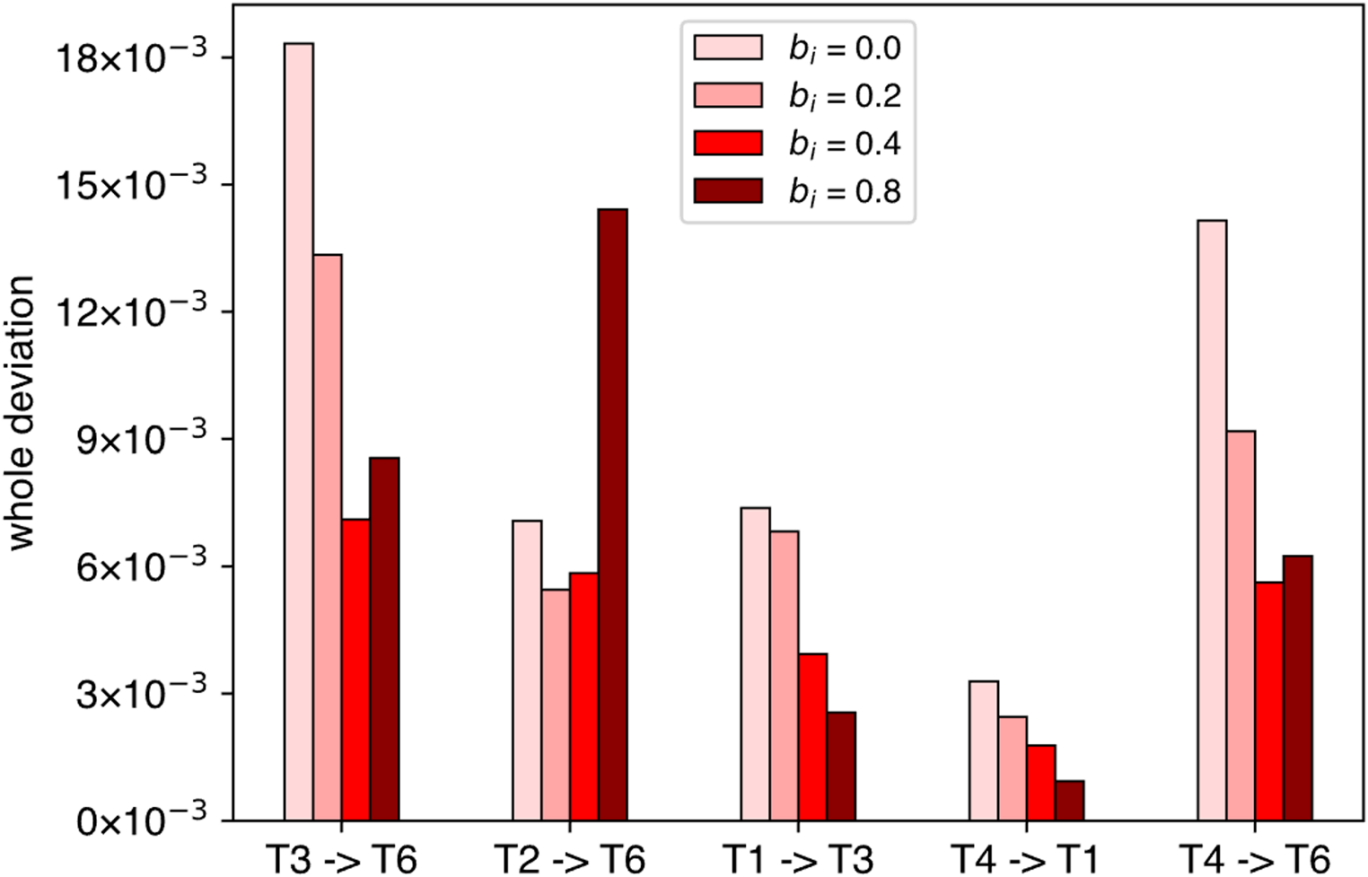
Comparison of the whole deviations of all trajectories shown in Fig. 4

Figure 4 displays the five minimum torque-change trajectories when all Winter’s biomechanically relevant physical parameters shown in Table 1 were simultaneously incorporated. The whole deviation corresponding to each trajectory is shown in Fig. 5. Under low viscosity conditions (${b}_{i}=0.0,\:0.2,\:0.4$ N m s/rad), the trajectories tended to be concave or curved leftward relative to the original trajectories. The velocity profiles also tended to be skewed. As the viscosity increased (${b}_{i}=0.8$ N m s/rad), these tendencies toward curvature and skewness became less pronounced. However, the directions of curvature (rightward or leftward) varied across five trajectories. Even with substantial viscosity (${b}_{i}=0.4,\:0.8$ N m s/rad), the trajectories did not precisely overlap with the original straight-forward ones, exhibiting some degree of curvature deviation (mean absolute change in whole deviation relative to Uno’s baseline: 100.8% for ${b}_{i}=0.4$ N m s/rad and 93.1% for ${b}_{i}=0.8$ N m s/rad across the five trajectories). This suggests that modifying viscosity within this higher range does not restore the trajectories to the original form.    

**Fig. 6:**
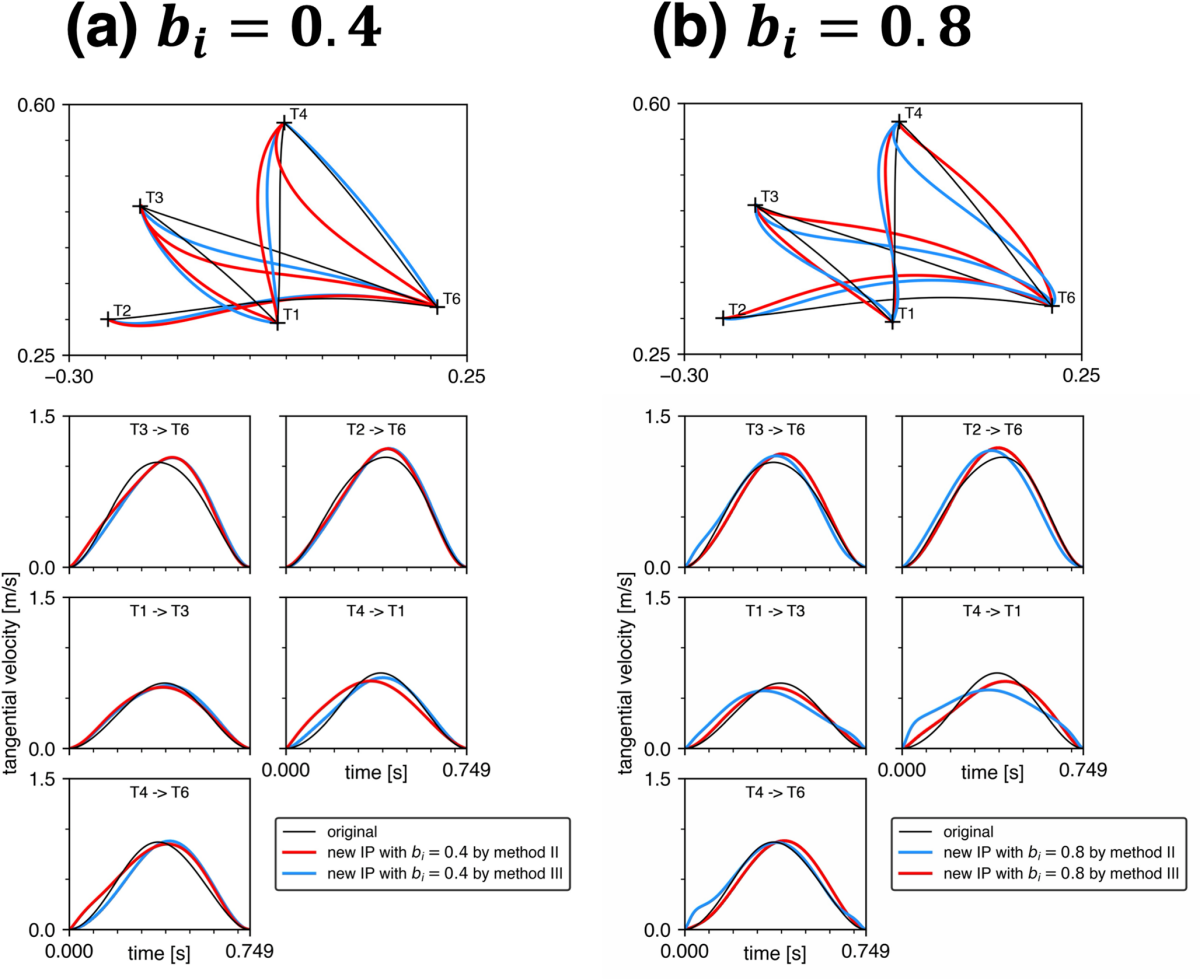
Comparisons of the minimum torque-change trajectories predicted by Uno’s original model (black) with those predicted when all inertial parameters (IP) were modified according to Winter’s biomechanically relevant physical parameters. The modified trajectories were calculated using two methods, II and III. Trajectories with smaller objective function values are shown in red, while those with larger values are shown in cyan. The viscous coefficients ${b}_{1}$ and ${b}_{2}$ were equal and set as 0.4 or 0.8. Comparisons of the hand tangential velocities of the five trajectories are also included

**Fig. 7:**
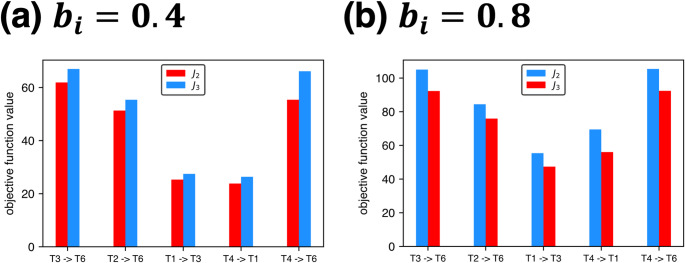
Comparison of the calculated values of the objective functions ${J}_{2}$ and ${J}_{3}$ obtained using method II and III, respectively, for all five trajectories shown in Fig. 6

As described in the previous section, the minimum torque-change trajectories for ${b}_{i}=0.4$ and $0.8$ N m s/rad in Fig. 4 were selected from the results of the two annealing methods (II and III) based on the criterion of smaller objective function $J$ values. The alternative trajectories not selected here are shown in Fig. 6. Figure 7 compares the specific values of the objective function ${J}_{2}$ and ${J}_{3}$ for methods II and III, respectively, across all five trajectories. The differences in objective function values between the two methods were minimal. Importantly, the trajectories that appear closer to the original straight paths are not necessarily optimal in terms of minimizing cumulative torque changes. Together, these results indicate that trajectories that appear closer to straight paths are not necessarily those that minimize cumulative torque change, and that the predicted trajectory shape may depend on which local optimum is reached.

## Discussions

This study showed that the minimum torque-change trajectories were largely affected and, hence, sensitive to changes in the mass and moment of inertia of the distal segment (forearm) (Fig. 2a, b). The reason is that the change in the rotational inertia around the distal joint should affect the rotational inertia around the proximal joint. In contrast, the change in the rotational inertia of the proximal segment does not affect the rotational inertia of the distal joint. Therefore, the equation of motion that the optimization process must satisfy as a constraint (Eq. 3) is more affected by the parameter change in the distal segment, possibly resulting in more curved hand trajectories. In addition, the minimum torque-change trajectories were also largely affected and, hence, sensitive to the increase in the viscosity in the proximal joint (shoulder) (Fig. 2c), possibly because the range of joint motion and, hence, the angular velocity of the shoulder joint tended to be large in the five reaching trajectories studied. It is thought that sensitivity increases as the elbow angle ${\theta}_{2}$ approaches singularity (0 or π rad). However, in this study, the observed trajectory curvature was not confined to the neighborhoods of a specific target, indicating that proximity to singular postures is unlikely to be the primary cause of the significant trajectory alterations. Thus, beyond the qualitative observation that the minimum torque-change model can yield curved trajectories, the present results provide a parameter-wise characterization of which biomechanical parameters most strongly drive deviations from straight hand paths.

In general, the hand trajectories of human point-to-point reaching movements are stereotypical and consistent within and across subjects (Morasso 1981). The present finding contradicts this observation in that the minimum torque-change trajectories are sensitive to the change in the arm’s physical parameters. This is because the arm’s physical parameters should vary substantially from person to person, and large changes in the arm’s parameters should substantially vary the results, possibly more curved hand trajectories in human reaching movements. The strength of the minimum torque-change model is the predictive power of roughly straight trajectories with bell-shaped velocity profiles that are stereotypically observed in actual human reaching movements (Uno et al. 1989). However, based on our results, the capability of predicting roughly straight trajectories might not be due to a general principle of the minimum torque-change model. Instead, it emerged due to a specific set of arm parameters determined in the original model.

The physical parameters of the arm used in the original model (Uno et al. 1989) are not necessarily anthropometrically relevant. Therefore, we recalculated the trajectories using a biomechanically more relevant set of parameters to investigate the possibility that the relevant set of parameters could reproduce human-like hand trajectories. However, this was not the case, as shown in Fig. 4. Therefore, the present study highlights a limitation of the minimum torque-change model and the necessity for further research. One limitation of the present study is the lack of sufficient empirical data and references that could serve as measures of intersubject variability (e.g., standard deviations) in the arm’s physical parameters and overall trajectory deviations. This limitation makes it difficult to directly compare the present simulation results with the variability observed in the general population. Resolving this issue by employing parameter recovery methods to reliably estimate actual parameter changes from noisy kinematic data will be crucial for further justifying the scaling of physical parameter modifications. In addition, robustness mapping to identify regions of the parameter space that can still generate roughly straight trajectories, as observed under the original parameters, will also be essential to determine whether the minimum torque-change model can remain useful through parameter tuning or whether it fundamentally lacks validity as a criterion for movement trajectory planning.

Our results suggest that models less sensitive to variations in the arm’s physical parameters should be more plausible frameworks for describing actual human reaching movements. Other intrinsic-dynamic models, such as the minimum commanded torque-change model (Nakano et al. 1999; Wada et al. 2001), should be examined for this sensitivity. Another candidate might be a trade-off between a dynamic criterion and an extrinsic-kinematic criterion, such as the minimum hand-jerk model, which predicts strictly straight trajectories. Moreover, within an optimal feedback control framework, the parameter sensitivity may be alleviated through adaptive feedback processes. Validating these various possibilities with respect to robustness to changes in physical parameters will also provide an important direction for future research.

Finally, the present study highlighted the difficulty of identifying the globally optimal minimum torque-change trajectory between a given start and end point. Even small differences in the cost function values can result in substantially different trajectories, and the outcomes are also sensitive to the choice of optimization method. Although using alternative approaches, such as the Euler–Poisson method (Wada et al. 2001), might provide a more stable optimization, it is still possible that multiple local minima would emerge, as in the present study. This issue represents a significant limitation that was not examined and should be addressed in future work. Nevertheless, given such unfavorable conditions, it is questionable whether the brain truly solves this type of problem. From this perspective as well, the minimum torque-change model does not appear to represent a biologically feasible computational framework for generating human reaching movements.

## Supplementary Information

Below is the link to the electronic supplementary material.

Supplementary Material 1

## Data Availability

No datasets were generated or analysed during the current study.

## Code availability

The Python and C++ source code used to simulate all data in this study is available from the authors, KM and TK, upon reasonable request.
