# Supplementary material for: Effect of changes in the arm physical parameters on the minimum torque-change trajectories of human reaching movements
Source: Cogn Neurodyn. 2026 Mar 4;20(1):59. doi: 10.1007/s11571-026-10428-0 (PMC12961019; doi:10.1007/s11571-026-10428-0)
Supplement: Supplementary file 1 — Supplementary Material 1 [file 11571_2026_10428_MOESM1_ESM.docx]

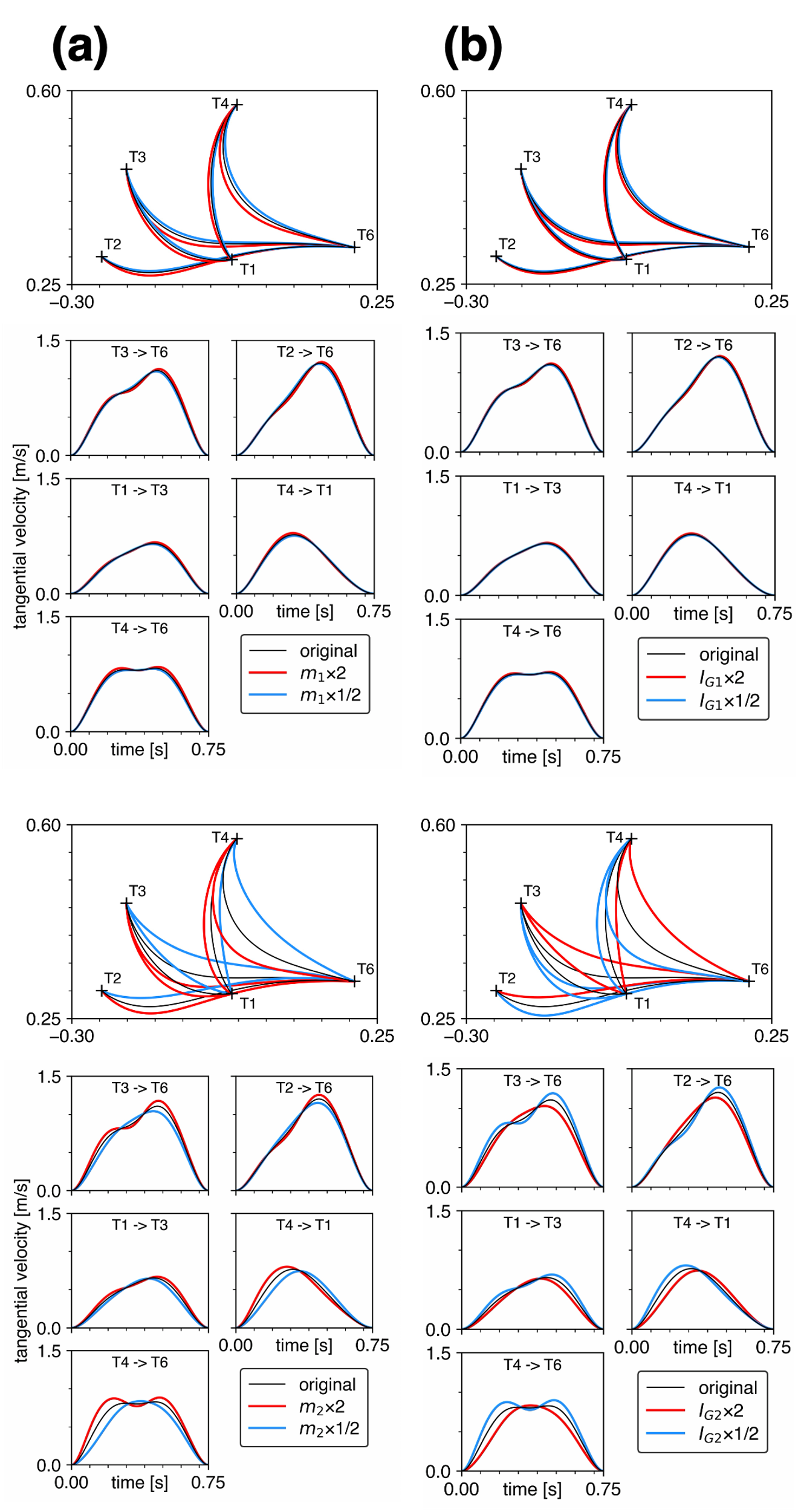


**Fig. S1** Comparisons of the minimum torque-change trajectories predicted by the baseline model using Winter’s parameter set (black) with those predicted when (a) the segment masses $m_{1}$ (top) and $m_{2}$ (bottom) were doubled (red) and the halved (cyan), (b) the segment moments of inertia $I_{G1}$ (top) and $I_{G2}$ (bottom) were doubled (red) or the halved (cyan), one-at-a-time. Note that $b_{1}$ and $b_{2}$​ were fixed at 0.08 N m s/rad in these simulations. Comparisons of the hand tangential velocities of the five trajectories are also included.

**Table S1** Whole deviations shown in Fig. 3. All values are in the order of $10⁻^{3}$.

|  |  | original |  | $m_{1}$ | |  | $m_{2}$ | |  | $I_{1}$ | |  | $I_{2}$ | |  | $b_{1}$ | |  | $b_{2}$ | |
| --- | --- | --- | --- | --- | --- | --- | --- | --- | --- | --- | --- | --- | --- | --- | --- | --- | --- | --- | --- | --- |
|  |  |  |  | $\times2$ | $\times1/2$ |  | $\times2$ | $\times1/2$ |  | $\times2$ | $\times1/2$ |  | $\times2$ | $\times1/2$ |  | $\times5$ | $\times1/5$ |  | $\times5$ | $\times1/5$ |
| T3 → T6 |  | 4.981 |  | 4.912 | 5.018 |  | 7.703 | 8.566 |  | 4.638 | 5.213 |  | 7.497 | 7.548 |  | 9.493 | 4.928 |  | 4.536 | 5.300 |
| T2 → T6 |  | 4.796 |  | 5.215 | 4.544 |  | 3.067 | 9.594 |  | 6.121 | 3.190 |  | 5.322 | 3.777 |  | 12.438 | 3.732 |  | 3.547 | 5.817 |
| T1 → T3 |  | 2.986 |  | 2.987 | 2.986 |  | 3.549 | 3.383 |  | 3.013 | 2.992 |  | 3.461 | 3.974 |  | 3.271 | 3.022 |  | 3.562 | 2.990 |
| T4 → T1 |  | 0.351 |  | 0.364 | 0.345 |  | 1.001 | 0.394 |  | 0.422 | 0.314 |  | 0.545 | 1.276 |  | 0.254 | 0.366 |  | 0.430 | 0.335 |
| T4 → T6 |  | 5.575 |  | 5.564 | 5.580 |  | 6.041 | 6.502 |  | 5.512 | 5.591 |  | 6.505 | 6.030 |  | 6.703 | 5.527 |  | 5.248 | 5.700 |

**Table S2** Whole deviations shown in Fig. 5. For the cases where $b_{i}=0.4$ and $0.8$, the whole deviations were also calculated for all trajectories calculated by both methods II and III. All values are in the order of $10⁻^{3}$.

|  |  | $b_{i}=0.0$  N m s/rad |  | $b_{i}=0.2$  N m s/rad |  | $b_{i}=0.4$  N m s/rad | |  | $b_{i}=0.8$  N m s/rad | |
| --- | --- | --- | --- | --- | --- | --- | --- | --- | --- | --- |
|  |  |  |  |  |  | II | III |  | II | III |
| T3 → T6 |  | 18.329 |  | 13.341 |  | 7.111 | 3.485 |  | 2.082 | 8.561 |
| T2 → T6 |  | 7.079 |  | 5.453 |  | 5.844 | 5.880 |  | 12.014 | 14.415 |
| T1 → T3 |  | 7.382 |  | 6.826 |  | 3.932 | 5.215 |  | 1.259 | 2.556 |
| T4 → T1 |  | 3.291 |  | 2.451 |  | 1.779 | 1.187 |  | 1.055 | 0.935 |
| T4 → T6 |  | 14.154 |  | 9.181 |  | 5.623 | 6.141 |  | 3.339 | 6.247 |

**Table S3** Calculated values of the objective functions $J_{2}$ and $J_{3}$ obtained using method II and III, respectively, shown in Fig. 7.

|  |  | $b_{i}=0.4$ N m s/rad | |  | $b_{i}=0.8$ N m s/rad | |
| --- | --- | --- | --- | --- | --- | --- |
|  |  | $J_{2}$ | $J_{3}$ |  | $J_{2}$ | $J_{3}$ |
| T3 → T6 |  | 61.90 | 66.99 |  | 105.03 | 92.30 |
| T2 → T6 |  | 51.32 | 55.38 |  | 84.43 | 75.94 |
| T1 → T3 |  | 25.31 | 27.46 |  | 55.39 | 47.38 |
| T4 → T1 |  | 23.83 | 26.37 |  | 69.48 | 56.06 |
| T4 → T6 |  | 55.40 | 66.13 |  | 105.41 | 92.41 |
